# *N*-(4-Methyl-2-nitro­phen­yl)succinamic acid

**DOI:** 10.1107/S1600536812007258

**Published:** 2012-02-24

**Authors:** U. Chaithanya, Sabine Foro, B. Thimme Gowda

**Affiliations:** a Department of Chemistry, Mangalore University, Mangalagangotri 574 199, Mangalore, India; b Institute of Materials Science, Darmstadt University of Technology, Petersenstrasse 23, D-64287 Darmstadt, Germany

## Abstract

In the title compound, C_11_H_12_N_2_O_5_, the conformation of the N—H bond in the amide segment is *syn* to the *ortho*-nitro group in the benzene ring. The amide C=O and the carboxyl C=O of the acid segment are *syn* to each other and both are *anti* to the H atoms on the adjacent –CH_2_ groups. Furthermore, the C=O and O—H bonds of the acid group are in *syn* positions with respect to each other. The dihedral angle between the benzene ring and the amide group is 36.1 (1)°. The amide H atom shows bifurcated intra­molecular hydrogen bonding with an O atom of the *ortho*-nitro group and an inter­molecular hydrogen bond with the carbonyl O atom of another mol­ecule. In the crystal, the N—H⋯O(C) hydrogen bonds generate a chain running along the [100] direction. Inversion dimers are formed *via* a pair of O—H⋯O(C) interactions, that form an eight-membered hydrogen-bonded ring involving the carboxyl group.

## Related literature

For our studies on the effects of substituents on the structures and other aspects of *N*-(ar­yl)-amides, see: Gowda *et al.* (1999 ▶, 2006 ▶); Chaithanya *et al.* (2012 ▶). For *N*-(ar­yl)-methane­sulfon­amides, see: Gowda *et al.* (2007 ▶). For *N*-chloro­aryl­amides, see: Jyothi & Gowda (2004 ▶). For *N*-bromo­aryl­sulfonamides, see: Usha & Gowda (2006 ▶).
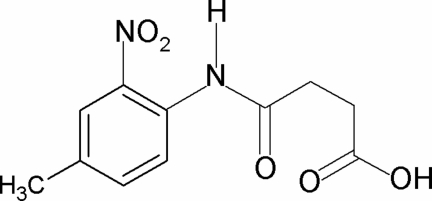


## Experimental

### 

#### Crystal data

C_11_H_12_N_2_O_5_
*M* *_r_* = 252.23
Triclinic, 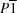

*a* = 4.8531 (7) Å
*b* = 11.015 (2) Å
*c* = 11.787 (2) Å
α = 69.59 (1)°
β = 78.77 (1)°
γ = 83.62 (2)°
*V* = 578.59 (17) Å^3^
*Z* = 2
Mo *K*α radiation
μ = 0.12 mm^−1^
*T* = 293 K
0.40 × 0.22 × 0.12 mm

#### Data collection

Oxford Diffraction Xcalibur diffractometer with a Sapphire CCD detector
Absorption correction: multi-scan (*CrysAlis RED*; Oxford Diffraction, 2009 ▶) *T*_min_ = 0.955, *T*_max_ = 0.986
3557 measured reflections
2314 independent reflections
1900 reflections with *I* > 2σ(*I*)
*R*_int_ = 0.011

#### Refinement

*R*[*F* ^2^ > 2σ(*F* ^2^)] = 0.046
*wR*(*F* ^2^) = 0.119
*S* = 1.05
2314 reflections
170 parameters
2 restraints
H atoms treated by a mixture of independent and constrained refinement
Δρ_max_ = 0.23 e Å^−3^
Δρ_min_ = −0.22 e Å^−3^

### 

Data collection: *CrysAlis CCD* (Oxford Diffraction, 2009 ▶); cell refinement: *CrysAlis CCD*; data reduction: *CrysAlis RED* (Oxford Diffraction, 2009 ▶); program(s) used to solve structure: *SHELXS97* (Sheldrick, 2008 ▶); program(s) used to refine structure: *SHELXL97* (Sheldrick, 2008 ▶); molecular graphics: *PLATON* (Spek, 2009 ▶); software used to prepare material for publication: *SHELXL97*.

## Supplementary Material

Crystal structure: contains datablock(s) I, global. DOI: 10.1107/S1600536812007258/kp2388sup1.cif

Structure factors: contains datablock(s) I. DOI: 10.1107/S1600536812007258/kp2388Isup2.hkl

Supplementary material file. DOI: 10.1107/S1600536812007258/kp2388Isup3.cml

Additional supplementary materials:  crystallographic information; 3D view; checkCIF report

## Figures and Tables

**Table 1:** Hydrogen-bond geometry (Å, °)

*D*—H⋯*A*	*D*—H	H⋯*A*	*D*⋯*A*	*D*—H⋯*A*
N1—H1N⋯O5	0.84 (2)	2.11 (2)	2.648 (2)	121 (2)
N1—H1N⋯O1^i^	0.84 (2)	2.34 (2)	3.072 (2)	146 (2)
O3—H3O⋯O2^ii^	0.83 (2)	1.86 (2)	2.688 (2)	176 (3)

Symmetry codes: (i) 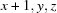; (ii) 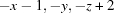.

**Fig. 1.**
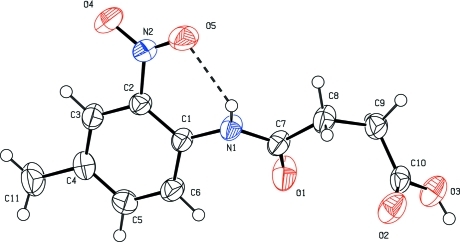
Molecular structure of the title compound showing the atom labelling scheme and intramolecular N-H···O hydrogen bond. Displacement ellipsoids are drawn at the 50% probability level.

**Fig. 2.**
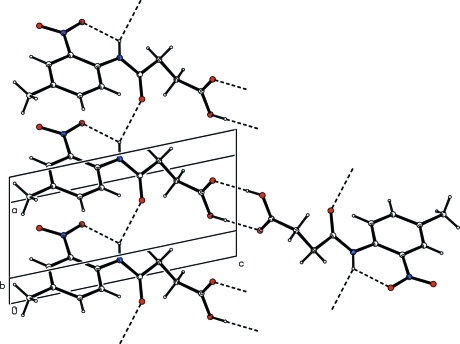
Molecular packing of the title compound with hydrogen bonding shown as dashed lines.

## supplementary crystallographic information

### Comment

As part of our studies on the substituent effects on the structures and other aspects of *N*-(aryl)-amides (Gowda *et al.*, 1999, 2006; Chaithanya *et al.*, 2012), *N*-(aryl)-methanesulfonamides (Gowda *et al.*, 2007), *N*-chloroarylsulfonamides (Jyothi & Gowda, 2004) and *N*-bromoarylsulfonamides (Usha & Gowda, 2006), in the present work, the crystal structure of *N*-(2-nitro-4-methylphenyl)succinamic acid has been determined (Fig. 1). The conformation of the N—H bond in the amide segment is *syn* to the *ortho*–nitro group in the benzene ring, similar to that observed between the N—H bond and the *ortho*–chloro atom in *N*-(2-chloro-4-methylphenyl)- succinamic acid (I) (Chaithanya *et al.*, 2012).

Further, the conformations of the amide oxygen and the carboxyl oxygen of the acid segment are *syn* to each other, in contrast to the *anti* conformation observed in (I). But both the amide oxygen and the carboxyl oxygen are *anti* to the H atoms on the adjacent –CH_2_ group, in both the compounds.

The dihedral angle between the phenyl ring and the amide group is 36.1 (1)°, compared to the value of 48.4 (1)° in (I). The amide H-atom shows bifurcated intramolecular H-bonding with the O-atom of the *ortho*-nitro group and the intermolecular H-bonding with the carbonyl oxygen atom of the other molecule. In the crystal, the molecules are packed into chains through intermolecular N—H···O(C) along the direction [100] and pairs of centrosymmetric O–H···O(C) hydrogen bonds (Table 1, Fig. 2).

### Experimental

The solution of succinic anhydride (0.01 mol) in toluene (25 mL) was treated dropwise with the solution of 2-nitro,4-methylaniline (0.01 mol) also in toluene (20 mL) with constant stirring. The resulting mixture was stirred for about one h and set aside for an additional h at room temperature for completion of the reaction. The mixture was then treated with dilute hydrochloric acid to remove the unreacted 2-nitro-4-methyl- aniline. The resultant title compound was filtered under suction and washed thoroughly with water to remove the unreacted succinic anhydride and succinic acid. Repeated recrystallisations from ethanol were applied until the constant melting point. The purity of the compound was checked and characterised by its infrared and NMR spectra.

Needle like yellow single crystals used in X-ray diffraction studies were grown in ethanolic solution by slow evaporation of the solvent at room temperature.

### Refinement

The H atoms of the NH group and the OH group were located in a difference map and later restrained to the distance N—H = 0.86 (2) Å and O—H = 0.82 (2) Å, respectively. The other H atoms were positioned with idealized geometry using a riding model with the aromatic C—H = 0.93 Å and methylene C—H = 0.97 Å. All H atoms were refined with isotropic displacement parameters set at 1.2 *U*_eq_(C-aromatic, N) and 1.5 *U*_eq_(*C*-methyl).

### Crystal data

**Table:**

C_11_H_12_N_2_O_5_	*Z* = 2
*M**_r_* = 252.23	*F*(000) = 264
Triclinic, *P*1	*D*_x_ = 1.448 Mg m^−^^3^
Hall symbol: -P 1	Mo *K*α radiation, λ = 0.71073 Å
*a* = 4.8531 (7) Å	Cell parameters from 1681 reflections
*b* = 11.015 (2) Å	θ = 3.1–27.8°
*c* = 11.787 (2) Å	µ = 0.12 mm^−^^1^
α = 69.59 (1)°	*T* = 293 K
β = 78.77 (1)°	Needle, yellow
γ = 83.62 (2)°	0.40 × 0.22 × 0.12 mm
*V* = 578.59 (17) Å^3^	

### Data collection

**Table:**

Oxford Diffraction Xcalibur diffractometer with a Sapphire CCD detector	2314 independent reflections
Radiation source: fine-focus sealed tube	1900 reflections with *I* > 2σ(*I*)
Graphite monochromator	*R*_int_ = 0.011
Rotation method data acquisition using ω and phi scans	θ_max_ = 26.4°, θ_min_ = 3.1°
Absorption correction: multi-scan (*CrysAlis RED*; Oxford Diffraction, 2009)	*h* = −5→6
*T*_min_ = 0.955, *T*_max_ = 0.986	*k* = −13→13
3557 measured reflections	*l* = −14→14

### Refinement

**Table:**

Refinement on *F*^2^	Primary atom site location: structure-invariant direct methods
Least-squares matrix: full	Secondary atom site location: difference Fourier map
*R*[*F*^2^ > 2σ(*F*^2^)] = 0.046	Hydrogen site location: inferred from neighbouring sites
*wR*(*F*^2^) = 0.119	H atoms treated by a mixture of independent and constrained refinement
*S* = 1.05	*w* = 1/[σ^2^(*F*_o_^2^) + (0.0471*P*)^2^ + 0.2987*P*] where *P* = (*F*_o_^2^ + 2*F*_c_^2^)/3
2314 reflections	(Δ/σ)_max_ = 0.006
170 parameters	Δρ_max_ = 0.23 e Å^−^^3^
2 restraints	Δρ_min_ = −0.22 e Å^−^^3^

### Special details

**Table:**

Experimental. CrysAlis RED (Oxford Diffraction, 2009) Empirical absorption correction using spherical harmonics, implemented in SCALE3 ABSPACK scaling algorithm.
Geometry. All e.s.d.'s (except the e.s.d. in the dihedral angle between two l.s. planes) are estimated using the full covariance matrix. The cell e.s.d.'s are taken into account individually in the estimation of e.s.d.'s in distances, angles and torsion angles; correlations between e.s.d.'s in cell parameters are only used when they are defined by crystal symmetry. An approximate (isotropic) treatment of cell e.s.d.'s is used for estimating e.s.d.'s involving l.s. planes.
Refinement. Refinement of *F*^2^ against ALL reflections. The weighted *R*-factor *wR* and goodness of fit *S* are based on *F*^2^, conventional *R*-factors *R* are based on *F*, with *F* set to zero for negative *F*^2^. The threshold expression of *F*^2^ > σ(*F*^2^) is used only for calculating *R*-factors(gt) *etc*. and is not relevant to the choice of reflections for refinement. *R*-factors based on *F*^2^ are statistically about twice as large as those based on *F*, and *R*- factors based on ALL data will be even larger.

### Fractional atomic coordinates and isotropic or equivalent isotropic displacement parameters (Å^2^)

**Table:**

	*x*	*y*	*z*	*U*_iso_*/*U*_eq_	
C1	0.0723 (3)	0.37100 (17)	0.38806 (15)	0.0344 (4)	
C2	0.2308 (4)	0.40404 (17)	0.26912 (16)	0.0351 (4)	
C3	0.1566 (4)	0.51144 (18)	0.17381 (17)	0.0423 (4)	
H3	0.2650	0.5299	0.0960	0.051*	
C4	−0.0749 (4)	0.59120 (18)	0.19244 (18)	0.0439 (5)	
C5	−0.2278 (4)	0.56114 (19)	0.31041 (19)	0.0463 (5)	
H5	−0.3823	0.6146	0.3257	0.056*	
C6	−0.1571 (4)	0.45416 (19)	0.40568 (17)	0.0429 (4)	
H6	−0.2652	0.4373	0.4834	0.052*	
C7	−0.0488 (4)	0.18197 (18)	0.57398 (16)	0.0385 (4)	
C8	0.0872 (4)	0.0644 (2)	0.65902 (18)	0.0474 (5)	
H8A	0.1761	0.0914	0.7127	0.057*	
H8B	0.2330	0.0268	0.6106	0.057*	
C9	−0.1198 (4)	−0.03824 (19)	0.73683 (18)	0.0459 (5)	
H9A	−0.2447	−0.0471	0.6855	0.055*	
H9B	−0.0163	−0.1206	0.7655	0.055*	
C10	−0.2937 (4)	−0.01095 (18)	0.84541 (16)	0.0392 (4)	
C11	−0.1609 (5)	0.7055 (2)	0.0885 (2)	0.0627 (6)	
H11A	−0.3126	0.6828	0.0593	0.075*	
H11B	−0.0037	0.7291	0.0228	0.075*	
H11C	−0.2211	0.7774	0.1169	0.075*	
N1	0.1378 (3)	0.26122 (16)	0.48575 (14)	0.0402 (4)	
H1N	0.308 (3)	0.237 (2)	0.485 (2)	0.048*	
N2	0.4831 (3)	0.32707 (15)	0.23859 (14)	0.0422 (4)	
O1	−0.3032 (3)	0.20209 (15)	0.58421 (14)	0.0599 (5)	
O2	−0.2182 (3)	0.05642 (16)	0.89554 (14)	0.0591 (4)	
O3	−0.5284 (3)	−0.07162 (16)	0.88538 (13)	0.0533 (4)	
H3O	−0.606 (5)	−0.063 (3)	0.9514 (18)	0.064*	
O4	0.5972 (4)	0.35547 (18)	0.13202 (15)	0.0802 (6)	
O5	0.5750 (3)	0.23852 (15)	0.31947 (14)	0.0579 (4)	

### Atomic displacement parameters (Å^2^)

**Table:**

	*U*^11^	*U*^22^	*U*^33^	*U*^12^	*U*^13^	*U*^23^
C1	0.0299 (8)	0.0388 (9)	0.0306 (8)	−0.0020 (7)	−0.0043 (7)	−0.0073 (7)
C2	0.0333 (9)	0.0362 (9)	0.0355 (9)	−0.0031 (7)	−0.0022 (7)	−0.0129 (7)
C3	0.0483 (11)	0.0408 (10)	0.0327 (9)	−0.0069 (8)	−0.0023 (8)	−0.0070 (8)
C4	0.0483 (11)	0.0377 (10)	0.0411 (10)	−0.0042 (8)	−0.0115 (8)	−0.0048 (8)
C5	0.0402 (10)	0.0427 (10)	0.0497 (11)	0.0075 (8)	−0.0062 (9)	−0.0114 (9)
C6	0.0361 (10)	0.0485 (11)	0.0363 (9)	0.0034 (8)	0.0006 (8)	−0.0096 (8)
C7	0.0297 (9)	0.0478 (10)	0.0306 (8)	0.0016 (7)	−0.0045 (7)	−0.0053 (8)
C8	0.0317 (9)	0.0548 (12)	0.0379 (10)	0.0051 (8)	−0.0017 (7)	0.0021 (9)
C9	0.0420 (10)	0.0419 (10)	0.0411 (10)	0.0059 (8)	−0.0029 (8)	−0.0026 (8)
C10	0.0329 (9)	0.0389 (9)	0.0347 (9)	0.0001 (7)	−0.0070 (7)	0.0016 (8)
C11	0.0699 (15)	0.0489 (12)	0.0536 (13)	0.0014 (11)	−0.0153 (11)	0.0036 (10)
N1	0.0255 (7)	0.0483 (9)	0.0344 (8)	0.0038 (6)	−0.0019 (6)	−0.0017 (7)
N2	0.0416 (9)	0.0422 (9)	0.0398 (8)	−0.0020 (7)	0.0036 (7)	−0.0154 (7)
O1	0.0268 (7)	0.0661 (10)	0.0599 (9)	0.0007 (6)	−0.0064 (6)	0.0112 (7)
O2	0.0535 (9)	0.0721 (10)	0.0508 (9)	−0.0233 (8)	0.0059 (7)	−0.0212 (8)
O3	0.0425 (8)	0.0697 (10)	0.0441 (8)	−0.0172 (7)	0.0014 (6)	−0.0150 (7)
O4	0.0901 (13)	0.0767 (12)	0.0462 (9)	0.0173 (10)	0.0240 (9)	−0.0119 (8)
O5	0.0500 (8)	0.0649 (10)	0.0499 (8)	0.0201 (7)	−0.0043 (7)	−0.0172 (8)

### Geometric parameters (Å, º)

**Table:**

C1—C6	1.392 (2)	C8—C9	1.516 (3)
C1—N1	1.405 (2)	C8—H8A	0.9700
C1—C2	1.406 (2)	C8—H8B	0.9700
C2—C3	1.388 (2)	C9—C10	1.494 (3)
C2—N2	1.469 (2)	C9—H9A	0.9700
C3—C4	1.379 (3)	C9—H9B	0.9700
C3—H3	0.9300	C10—O2	1.219 (2)
C4—C5	1.388 (3)	C10—O3	1.306 (2)
C4—C11	1.506 (3)	C11—H11A	0.9600
C5—C6	1.380 (3)	C11—H11B	0.9600
C5—H5	0.9300	C11—H11C	0.9600
C6—H6	0.9300	N1—H1N	0.840 (15)
C7—O1	1.220 (2)	N2—O4	1.215 (2)
C7—N1	1.356 (2)	N2—O5	1.216 (2)
C7—C8	1.510 (2)	O3—H3O	0.828 (17)
			
C6—C1—N1	120.65 (16)	C9—C8—H8B	109.0
C6—C1—C2	116.32 (16)	H8A—C8—H8B	107.8
N1—C1—C2	123.04 (16)	C10—C9—C8	114.65 (18)
C3—C2—C1	121.65 (16)	C10—C9—H9A	108.6
C3—C2—N2	116.31 (15)	C8—C9—H9A	108.6
C1—C2—N2	122.05 (15)	C10—C9—H9B	108.6
C4—C3—C2	121.19 (17)	C8—C9—H9B	108.6
C4—C3—H3	119.4	H9A—C9—H9B	107.6
C2—C3—H3	119.4	O2—C10—O3	123.11 (18)
C3—C4—C5	117.44 (17)	O2—C10—C9	123.32 (17)
C3—C4—C11	121.31 (19)	O3—C10—C9	113.48 (18)
C5—C4—C11	121.24 (19)	C4—C11—H11A	109.5
C6—C5—C4	121.87 (18)	C4—C11—H11B	109.5
C6—C5—H5	119.1	H11A—C11—H11B	109.5
C4—C5—H5	119.1	C4—C11—H11C	109.5
C5—C6—C1	121.50 (17)	H11A—C11—H11C	109.5
C5—C6—H6	119.3	H11B—C11—H11C	109.5
C1—C6—H6	119.3	C7—N1—C1	126.29 (15)
O1—C7—N1	123.78 (16)	C7—N1—H1N	116.4 (15)
O1—C7—C8	122.49 (16)	C1—N1—H1N	116.8 (15)
N1—C7—C8	113.73 (15)	O4—N2—O5	121.64 (17)
C7—C8—C9	113.01 (16)	O4—N2—C2	118.41 (17)
C7—C8—H8A	109.0	O5—N2—C2	119.95 (15)
C9—C8—H8A	109.0	C10—O3—H3O	110.5 (18)
C7—C8—H8B	109.0		
			
C6—C1—C2—C3	−2.0 (3)	O1—C7—C8—C9	13.1 (3)
N1—C1—C2—C3	178.40 (17)	N1—C7—C8—C9	−166.36 (18)
C6—C1—C2—N2	178.13 (16)	C7—C8—C9—C10	−79.0 (2)
N1—C1—C2—N2	−1.5 (3)	C8—C9—C10—O2	−25.8 (3)
C1—C2—C3—C4	0.7 (3)	C8—C9—C10—O3	157.49 (16)
N2—C2—C3—C4	−179.39 (17)	O1—C7—N1—C1	−5.4 (3)
C2—C3—C4—C5	1.0 (3)	C8—C7—N1—C1	174.13 (18)
C2—C3—C4—C11	−178.18 (19)	C6—C1—N1—C7	39.0 (3)
C3—C4—C5—C6	−1.4 (3)	C2—C1—N1—C7	−141.4 (2)
C11—C4—C5—C6	177.7 (2)	C3—C2—N2—O4	−5.4 (3)
C4—C5—C6—C1	0.1 (3)	C1—C2—N2—O4	174.42 (19)
N1—C1—C6—C5	−178.82 (18)	C3—C2—N2—O5	173.52 (17)
C2—C1—C6—C5	1.6 (3)	C1—C2—N2—O5	−6.6 (3)

### Hydrogen-bond geometry (Å, º)

**Table:**

*D*—H···*A*	*D*—H	H···*A*	*D*···*A*	*D*—H···*A*
N1—H1*N*···O5	0.84 (2)	2.11 (2)	2.648 (2)	121 (2)
N1—H1*N*···O1^i^	0.84 (2)	2.34 (2)	3.072 (2)	146 (2)
O3—H3*O*···O2^ii^	0.83 (2)	1.86 (2)	2.688 (2)	176 (3)

Symmetry codes: (i) *x*+1, *y*, *z*; (ii) −*x*−1, −*y*, −*z*+2.
